# Evaluation of cell membrane integrity as a potential antimicrobial target for plant products

**DOI:** 10.1186/1472-6882-14-278

**Published:** 2014-07-30

**Authors:** Tariro A Chitemerere, Stanley Mukanganyama

**Affiliations:** Biomolecular Interactions Analyses Laboratory, Department of Biochemistry, University of Zimbabwe, P.O MP 167, Mount Pleasant Harare, Zimbabwe

**Keywords:** Bacterial efflux pumps, Membrane permeability, *C. citrinus*, *V. adoensis*

## Abstract

**Background:**

There is urgent need to discover new antimicrobial compounds with diverse chemical structures and mechanisms of action due to increasing new and re-emerging infectious diseases. Additionally, appearance of undesirable side effects of certain antibiotics and increasing resistance to antibiotics in current clinical use is also a cause for concern. Bacterial cell membranes are a possible target for developing new antibacterial drugs since membrane-based efflux pump systems play an important role in bacterial pathogenicity and antimicrobial resistance in bacteria. Hence, the objective of our study was to evaluate bacterial membrane integrity of two species of bacteria; *Staphylococcus aureus* and *Pseudomonas aeruginosa*, in the presence of ethanolic leaf extracts of two plant species *Callistemon citrinus* and *Vernonia adoensis* from Zimbabwe.

**Methods:**

Bacterial efflux pump inhibition using both leaf extracts was determined by monitoring the transport of Rhodamine 6 G (R6G) across the cell membrane and IC_50_ values were obtained. Membrane permeabilizing properties of both extracts were also evaluated using the membrane potential sensitive dye 3’3 dipropylthiadicarbocyanine (diSC3-5). Haemolysis effect of both extracts on sheep erythrocytes was also investigated.

**Results:**

Both extracts inhibited bacterial efflux pumps which resulted in the accumulation of R6G inside the cell. The IC_50_ values for *C. citrinus* and *V.adoensis* against *S. aureus* were 1.44 mg/ml and 1.61 mg/ml, respectively. Both leaf extracts however, showed similar IC_50_ values of 1.64 mg/ml against *P. aeruginosa*. Both plant extracts showed some significant effects on permeability of the bacterial membrane when a 24-28% increase of diSC3-5 dye release was observed for *S. aureus* and 45–53% of dye was released from *P. aeruginosa* cell membrane after a 60 minute incubation period. In addition, both extracts exhibited haemolytic effects on sheep erythrocytes at concentrations greater than 2.5 mg/ml.

**Conclusions:**

These plant extracts may provide new lead compounds for developing potential efflux pump inhibitors (EPIs) or permeabilising agents that could aid the transport of antibacterial agents into bacterial cells.

## Background

Bacterial infections account for a significant proportion of the global infectious disease burden hence giving a negative impact on human welfare and the economy [1]. Morbidity and mortality rates caused by infectious microbial agents have been shown to pose serious public health concerns. Throughout the world, about 50–75% of hospital deaths were reported due to infectious diseases [2]. These numbers continue to increase due to development of resistance in microorganisms against the existing first line drugs. Thus, it is critical that a clear understanding of the biological aspects of infectious disease be made known so that eventually infectious disease morbidity and mortality are curtailed, if not eventually eradicated [3]. The human-mediated use and abuse of classical antibiotics has created a strong selective pressure for the rapid evolution of antibiotic resistance. As resistance levels rise, the efficacy of classical antibiotics wanes. Bacterial drug resistance is emerging as one of the most significant challenges to human health [4]. Multi-drug resistant microbial infections caused by Gram positive bacteria such as *Staphylococcus aureus* and *Enterococcus faecalis* represent an exponentially growing problem affecting communities worldwide [5].

Efflux pump-mediated resistance to single or multiple antimicrobial agents has not only raised serious concerns but also has constricted the treatment options against bacterial infections. Efflux pumps reduce the accumulation of antibiotics inside of the bacterial cells, and the slow phase in which the process of antibiotic efflux takes place provides sufficient time for the bacterium to adapt to the antibiotics and become resistant through mutations or alteration of antibiotic targets [3]. Due to the development of this resistance in human pathogens against commonly used antibiotics, it has become necessary to search for new antimicrobial substances from other sources including plants [6].

The use of medicinal plants as a source for relief from illness is doubtless an art as old as mankind [7]. Plants are the richest resource of drugs of traditional systems of medicine, modern medicines, nutraceuticals, food supplements, folk medicines, pharmaceutical intermediates and chemical entities for synthetic drugs [8]. Plants have been known to synthesize a variety of compounds to protect themselves against a variety of their own pathogens and, therefore, can be considered as potential source of different classes of antimicrobial substances [6].

*Callistemon citrinus* (Linn.), commonly known as ‘Crimson Bottle Brush’ , is an evergreen tree or shrub, belonging to the family Myrtaceae [7]. The species of this genus are mostly used for ornamental purpose. However, Callistemon spp. are also sources of insecticidal, antibacterial and antifungal bioactive compounds [9]. *Callistemon citrinus* grows up to 6–15 m in height and 1.3-1.5 m in girth with sharp pointed mid-green leaves [7]. The different parts of this herb have been used in common remedies for treatment of diarrhoea, dysentery and rheumatism. The plant is also used as a water accent, anti-cough, anti-bronchitis and insecticide in folk medicine. Phytochemically, the plant is rich in polyphenols and essential oils such as a-pinene, b-pinene, a-terpinene, 1, 8-cineole, linalool, trans-pinocarveol, terpinen-4-ol, geranioland a-terpineol that has showed antibacterial activities [7, 9]. *Vernonia adoensis* is used traditionally by many communities to treat various illnesses due to lack of resources to access hospitals or even preference of the use of medicinal plants. The plant roots are used mainly for the treatment of sexually transmitted gonorrhoea by people in the Rift valley and Western part of Kenya [10]. The plant leaves are used in the treatment of malaria. The decoction of the roots mixed with the bark of other trees is used in the treatment of heart and kidney problems. Another study showed very high anti-plasmodia activity and the leaves are used to treat T.B. Much research has not being done to test the phytochemical analysis of this plant [10].

Bacterial cell membranes can be used as a target for the development of new antibacterial drugs. Many plants produce secondary metabolites which contain a steroid or triterpernoid aglycon attached to one or more sugar chains that exhibit cell membrane permeabilizing properties [11]. Permeability enhancers are agents that decrease or remove extra cellular layer resistance reversibly and allow the drug to pass through. Hence there is a pivotal relationship between permeability and enhancement activity and toxicity of the drug since there is easy passage into the cell. Moreover, the permeability enhancing effect of surfactants such as saponins is not only related to their nature, but also depends on other characteristics like electrical charge, polarity and the membrane [11]. Evaluating the permeability of enhancers using biological membranes plays an important role.

Initial screening results showed that leaf extracts from *C. citrinus* and *V. adoensis* were potent antibacterials with bactericidal activities at low concentrations of 250 μg/ml and 500 μg/ml, respectively [12]. Furthermore, EPI properties were also displayed by both extracts, making them potential sources of lead compounds for the development of new antibacterial agents. Moreover, these findings provided a basis to further investigate the potential target for the plant extracts responsible for the antibacterial activities. In this current study, the bacterial membrane was investigated as a potential antimicrobial target. The concentration required for 50% inhibition (IC_50_) of efflux activity in *S. aureus* and *P. aeruginosa* was determined for both plant extracts by monitoring the transport of Rhodamine 6 G across the cell membrane. Furthermore, the cytoplasmic membrane permeabilising properties of the extracts were also investigated. Plant compounds that could permeabilise bacterial membranes would be useful in the fight against bacterial infections since a weakened bacterial membrane would provide an easy passage of antibiotics into the bacterial cell. Finally, membrane haemolysing properties of the extracts were tested on sheep erythrocytes in order to determine the concentrations of the extracts that are safe to use without being toxic to the human body.

## Methods

### Plant material

*C. citrinus* was collected at the University of Zimbabwe campus in Harare province and *V. adoensis* was collected from Mashonaland Central (Centenary). This current study is a follow up on the previously reported antibacterial activity of the two extracts. The authentication and voucher specimen deposits were as reported in Chitemerere and Mukanganyama [12].

### Preparation of extracts

The preparation of plant extracts was described by Chitemerere and Mukanganyama [12]. Briefly, plant samples were ground in a two-speed blender (Cole Parmer Instrument Co., Vernon Hills, USA). A volume of 8 ml of ethanol was added to 2 g of grounded powder and shaken for 5 min on a vortex mixer and left for 10 min. The plant suspension was then transferred into a syringe and filtered into a small glass vial. The sterile suspension was filtered again using 0.45 μM Millipore sterile filter (Sigma-Aldrich, Taufkirchen, Germany) into a labelled small glass vial. Ethanol was left to evaporate overnight in fume hood with an air stream. A constant dry weight of each extract was obtained and the residues were stored at 4°C.

### Materials

All the chemicals, including ethanol, dimethylsufoxide (DMSO), rhodamine 6G (R6G), reserpine, 3,3’-dipropylthiadicarbocyanine DiSC_3_(5) iodide, phosphate buffered saline (PBS) and sodium azide (NaN_3_) were purchased from Sigma Chemical Co. (Taufkirchen, Germany) and all the solvents used were of analytical grade. Sheep erythrocytes were collected from the animal house at the University of Zimbabwe.

### Microorganisms and growth conditions

A Gram-positive and a Gram-negative bacterial strain; *Staphylococcus aureus* (ATCC 9144) and *Pseudomonas aeruginosa* (ATCC 27853), respectively were obtained from the Division of Microbiology, Department of Biological Sciences, University of Botswana. All strains were maintained in 50% glycerol in Eppendorf® microtubes and kept at −30°C until use. Prior to use, bacteria were grown in nutrient broth at 37°C for 24 h.

### Rhodamine 6G uptake

The concentration at which extracts of the two plant species inhibited the activity of bacterial efflux pumps by 50% (IC_50_) was determined in R6G accumulation experiments using the method of Maesaki et al. [13] with some modifications. Rhodamine 6 G is a fluorescent dye which is a substrate of efflux pumps and it is used a model for an antibiotic in this study. Bacteria were cultured overnight at 37°C at 110 rpm with constant shaking. After 24 h, cells were centrifuged using a Rotofix 32 centrifuge (Hettich Zentrifugen, Tuttlingen, Germany) at 4000 rpm for 5 min and washed twice with PBS (pH 7.2). Cells were centrifuged again and re-suspended at 40 mg/ml in PBS containing 10 mM NaN_3_. R6G was added to a final concentration of 10 μm and cells placed in an incubator for 1 h. Cells were centrifuged for 5 min at 4000 rpm and re-suspended in PBS containing 1 M glucose. The cells were divided into aliquots. Plant extract was then serially diluted in the range 3.84 mg/ml - 0 mg/ml and added to the cells. All tubes containing cells and the extracts at different concentrations were then placed in an incubator with agitation for 30 min at 37°C. Cells were centrifuged and the supernatant was discarded. The remaining pellet was re-suspended in 0.1 M glycine-HCl (pH 3) and placed in a shaking incubator overnight. After 24 h, cells were centrifuged for 10 min at 4000 rpm and the supernatant collected for measuring absorbance at 527 nm using an ELISA plate reader. Reserpine, a standard plant-based EPI, was used as a positive control.

### Cytoplasmic membrane permeability assay

The method of Nusslein et al. [4] was used to investigate the cytoplasmic membrane permeabilizing properties of the extracts of *C. citrinus* and *V. adoensis* against bacterial cell membranes using disC3-5. Bacterial cells were grown to mid-exponential phase and collected by centrifugation. Cells were washed once with buffer (5 mM HEPES, pH 7.2, 5 mM glucose) and re-suspend to 40 mg/ml in buffer. Cells were then incubated with 1 μM diSC3-5 for 1 hour, for maximal uptake of dye. Afterwards, 100 mM KCl was added to equilibrate the cytoplasmic and external K^+^ ion concentrations. Cells were then mixed with the desired concentration of the permeabilizing agent (plant extract 500 μg/ml) and the fluorescence was monitored at an excitation wavelength of 622 nm and an emission wavelength of 670 nm. Dye released with the addition of 1% DMSO was monitored as a positive control while ethanol was used as a negative control since the plant extracts were dissolved in ethanol. The monitoring period was for 1 hour at 10 minute intervals. During the assay, 750 μl of sample were withdrawn at 10 minute intervals and placed in eppendorf tubes with 750 μl of HEPES buffer. Samples were then centrifuged and the supernatants were collected for measuring fluorescence using the spectroflourophotometer (Shimadzu spectrofluorophotometer, RF-1501, Shimadzu Corporation, Kyoto, Japan).

### Haemolysis assay

Haemolysis assay was carried out as illustrated by Noudeh et al. [11]. Blood was collected from an adult sheep (University of Zimbabwe Animal House). This study was supported by the University of Zimbabwe Department of Biochemistry Research Ethics Commitee (01. 11. 11, UZBREC 02). Blood collected, was added to a 50 ml tube containing 11 mM sodium citrate and immediately mixed with an equal volume of Alsevier solution. Afterwards, 3 ml of the blood solution was centrifuged at 3000 rpm for 10 min and the supernatant was removed and the erythrocytes were washed three times in at least five times of their volume with McIlvaine’s buffer (pH 7.0). Following that, an erythrocyte suspension with 12% hematocrit was prepared and kept in 4°C prior to use. A suspension of erythrocytes (200 μl) was incubated with an equal volume of *C. citrinus* leaf extract or *V. adoensis* leaf extract for 1 hour within a micro-tube, at 25 and 37°C. After incubation, the mixture was centrifuged at 3000 rpm for 35 s and 200 μl of the resulting supernatant was added to 3 ml of Drabkin’s reagent. The amount of haemoglobin released against the samples was determined using an ELISA plate reader at 540 nm. Positive controls consisted of 200 μl of uncentrifuged erythrocyte suspension and 200 μl of McIlvaine’s buffer, which was added to 3 ml Drabkin’s reagent to obtain a value for 100% haemolysis. Additionally, a negative control, included to measure the level of spontaneous haemolysis, was comprised of 200 μl McIlvaine’s buffer mixed with 200 μl of the supernatant from centrifuged erythrocytes, and added to 3 ml of Drabkin’s reagent. Haemolysis percentage for each sample was calculated by dividing sample’s absorbance on positive control absorbance (complete haemolysis) multiplied by 100.

### Statistical analysis

One way ANOVA was used to analyse the results. All values are expressed as the mean ± standard deviation and *P* ≤ 0.05 values were considered to indicate statistically significant differences. Numerical data were analysed using the Student’s *t*-test using Graphpad™ version 5 for Windows (Graphpad™ Software Inc., San Diego, California, USA).

## Results

### Measurement of R6G uptake

**Figure 1:**
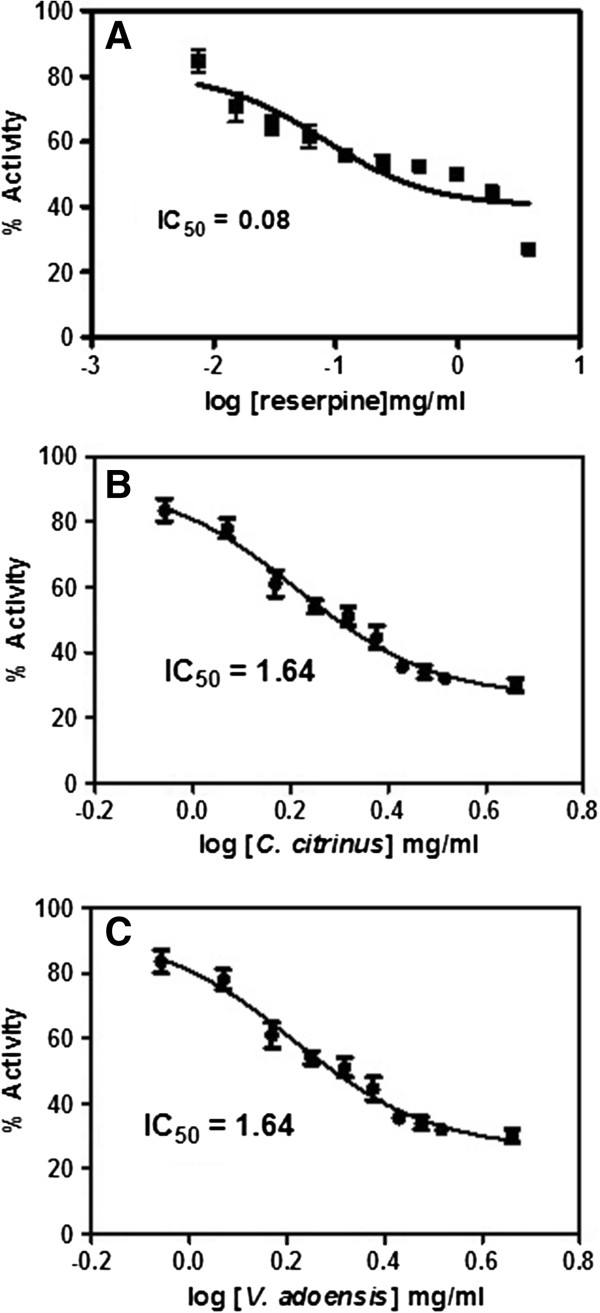
**R6G uptake and IC**_**50**_ **of efflux pump inhibition activity for A (reserpine), B** ***(C. citrinus)*** **and C (** ***V. adoensis)*** **against** ***P. aeruginosa.***

**Figure 2:**
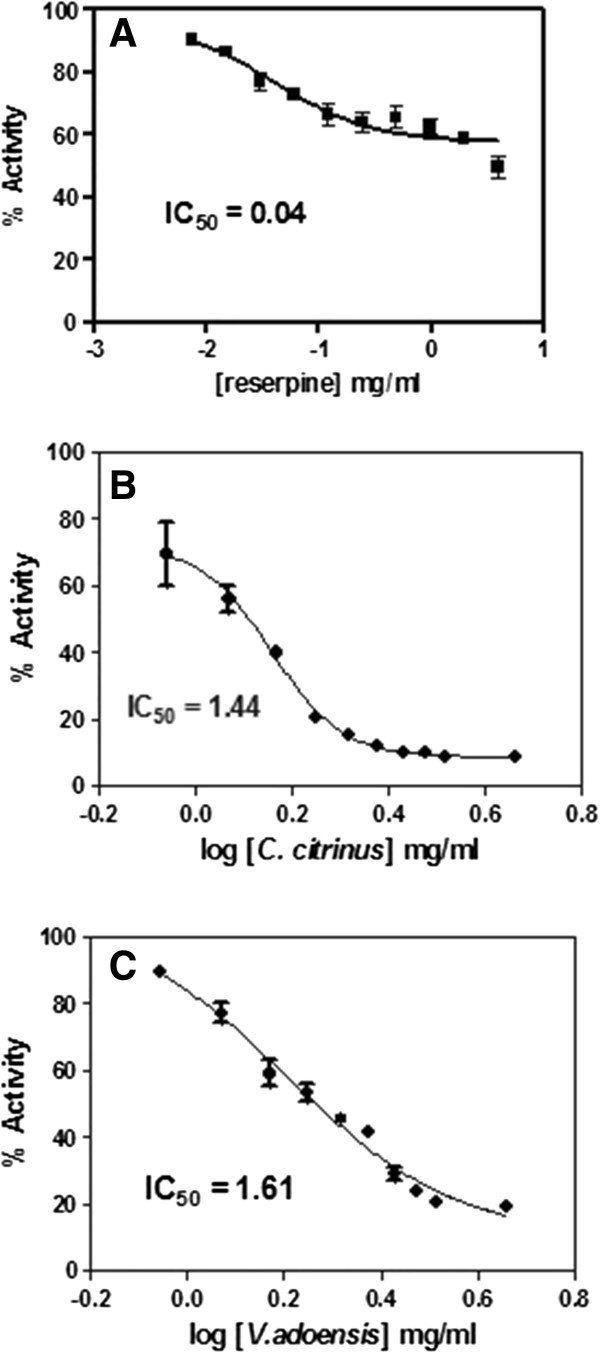
**R6G uptake and IC**_**50**_ **of efflux pump inhibition activity for A (reserpine), B (** ***C. citrinus)*** **and C (** ***V. adoensis)*** **against** ***S. aureus.***

As shown in Figures 1A and 2A, reserpine exhibited the lowest IC_50_ of 0.08 mg/ml and 0.04 mg/ml against *P. aeruginosa* and *S. aureus*, respectively. On the other hand, both extracts had similar EPI activity against *P. aeruginosa* with an IC_50_ of 1.64 mg/ml **(**Figure 1B and C). Following that, *C. citrinus* and *V. adoensis* leaf extracts displayed an IC_50_ of 1.44 mg/ml and 1.61 mg/ml against *S. aueus*, respectively. Although both plant extracts exhibited efflux pump inhibition, the activity was lower than the standard plant-based EPI, reserpine. These observations showed that the leaf extract of *C. citrinus* was more potent as an EPI than the *V. adoensis* leaf extract against *S. aureus*. Additionally, the EPI activity of both leaf extracts was comparable against *P. aeruginosa.*

### Cytoplasmic membrane permeability assay

**Figure 3:**
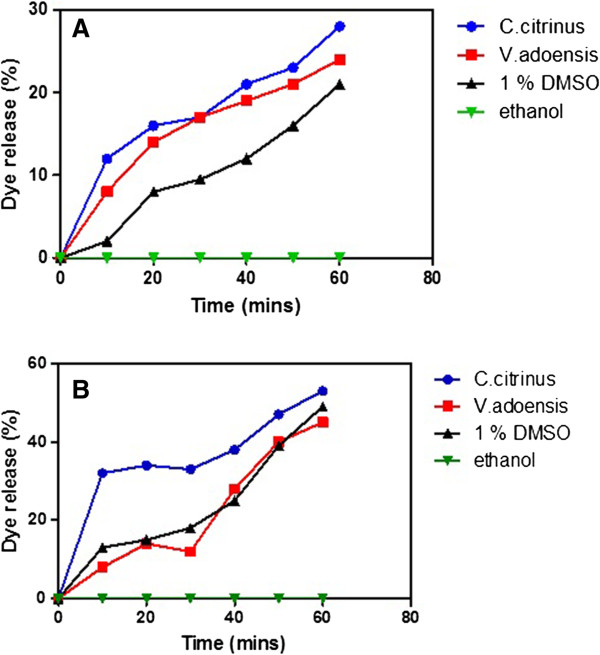
**Measurement of 3’3 dipropylthiadicarbocyanine (diSC3-5) dye release overtime from** ***S. aureus*** **(A) and** ***P. aeruginosa*** **(B) membranes in the presence of permeabilizing agent 1% DMSO, negative control ethanol and plant extracts** ***C. citrinus*** **and** ***V. adoensis*** **.**

Results from the cytoplasmic membrane permeability assay, showed a gradual increase of diSC3-5 dye release over time in the presence of both extracts of *C. citrinus* and *V. adoensis* as well as in the presence of 1% DMSO (Figure 3). At the end of a 60 minute monitoring period against *S. aureus*, a 28% dye release was observed for *C. citrinus* and 24% of the dye was release in the presence of *V. adoensis.* Furthermore, 21% of the diSC3-5 dye was release by 1% DMSO. However, both plant extracts were more potent as membrane permeability enhancers against *P. aeruginosa* as compared to *S. aureus*. This is shown by higher levels of dye released at 60 minutes for both *C. citrinus* (53%) and *V. adoensis* (45%). The *P. aeruginosa* membrane was also more sensitive to 1% DMSO as compared to *S. aueus* sensitivity, with a 49% dye release at 60 minutes (Table 1). There was no increase in dye release overtime in the presence of ethanol which was used a negative control. From the results there was more diSC3-5 released from the *P. aeriginosa* membrane as compared to the *S. aureus* membrane at the end of 60 minutes hence the Gram-negative membrane could be easily permeabilized as compared to the Gram-positive membrane for *S. aureus*. Overall, both *C. citrinus* and *V. adoensis* extracts showed bacterial membrane permeabilizing properties as shown by the increase in the percentage of dye leaking from the membrane with time.

**Table 1:** **Percentage increase of amount of dye being released from bacterial membranes in the presence of extracts of** ***C. citrinus*** **, for** ***V. adoensis*** **and DMSO after 60 mins**

Sample	% increase of dye released at 60 mins ***S. aureus***	% increase of dye released at 60 mins ***P. aeruginosa***
*C. citrinus*	28	53
*V. adoensis*	24	45
1% DMSO	21	49

### Haemolysis assay

**Figure 4:**
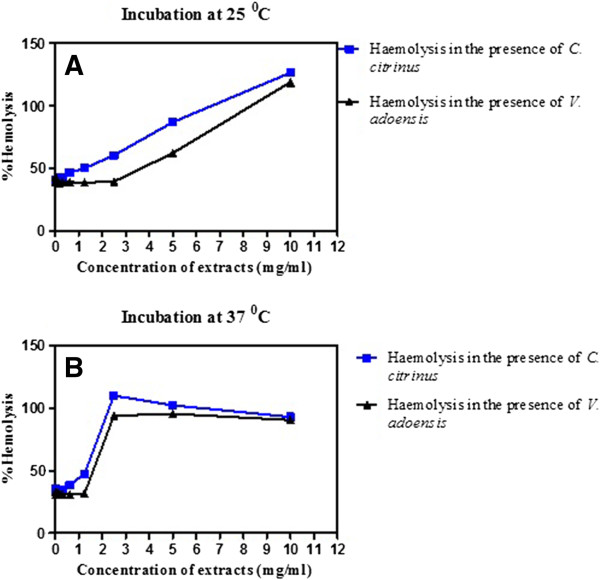
**Extent of haemolysis of sheep erythrocytes with increasing concentration of plant extracts at the end of a 90 minute incubation period at 25°C (A) and 37°C (B).**

After being exposed to increasing concentrations of the leaf extracts of *C. citrinus* and *V. adoensis* leaf extracts for a period of 90 min, there was an increase in the amount of haemolysis in two different incubation temperatures (Table 2). At 25°C, 39% haemolysis was observed up to a concentration of 2.5 mg/ml for *V. adoensis* (Figure 4). However, for concentrations greater than 2.5 mg/ml an increase in haemolysis as concentration increases is observed. For *C. citrinus,* less than 45% haemolysis was observed at concentrations less than 1 mg/ml. At 37°C, less than 40% haemolysis was observed at 1 mg/ml concentration of the leaf extract of *C. citrinus,* afterwhich complete haemolysis of 100% was obtained at 2 mg/ml concentrations. On the other hand, 29% haemolysis was observed for the leaf extract of *V. adoensis* at 1 mg/ml, at 37°C (Figure 4). However, rapid increase in haemolysis was detected at more than 1 mg/ml against both extracts at 37°C. In addition, both extracts displayed 100% haemolysis at concentration more than 2.5 mg/ml. Results also showed that the highest percentage of haemolysis was observed when the erythrocytes were incubated at 37°C as compared to at 25°C. Moreover, at 25°C and 37°C, both extracts concentration of lower than 1.5 mg/ml and 1 mg/ml did not harm the erythrocytes in an incubation period of 90 min, respectively.

**Table 2:** **Haemolysis at different concentrations of plant extract at the end of a 90 minute incubation at 25°C and 37°C**

Incubation temperature	Plant sample	% haemolysis 1 mg/ml	% haemolysis 2.5 mg/ml	% haemolysis 10 mg/ml
25°C	*C. citrinus*	47	60	124
	*V. adoensis*	36	37	119
37°C	*C. citrinus*	40	110	93
	*V. adoensis*	29	94	90

## Discussion

Inhibition of efflux pumps is increasingly becoming a way to fight MDR micro-organisms. In this study, the IC_50_ values of EPI activities for the two plant extracts against *S. aureus* and *P. aeruginosa* were determined. The IC_50_ values for the crude extracts were higher than the standard plant-based EPI, reserpine, which was used as the positive control. This is most likely because the crude extracts have a mixture of a lot of other phytochemicals which may be working antagonistically with the efflux pump inhibition properties of unidentified compound/s in the extracts. Further isolation and identification of EPI compound/s from *C. citrinus* and *V. adoensis* leaf extracts could increase the antibacterial activity. Many plants have been evaluated not only for their inherent antimicrobial activity, but also for their action as a resistant modifying agent (RMA) [14–16]. Some plant-derived EPIs are already well-known and used as standards such as reserpine from *Rauvolfia vomitoria* and piperine from *Piper nigrum* both of which are alkaloids [17, 18]. Several other plant-derived compounds, including the terpene carnosic acid from *Rosmarinus officinalis*, the diterpene totarol from *Chamaecyparis nootkatensis*, and the flavonolignan 5-methoxyhydnocarpin, inhibits NorA efflux pump activity in *S. aureus* and synergistically increases the activity of the antimicrobial alkaloid berberine present in the same plant [19]. Fiamegos et al. [5] and Garvey et al. [20] also demonstrated efflux pump inhibition activity from plants.

Hence, isolation of phytoconstituents from the plants could lead to potential EPIs comparable to the standard inhibitors. The inhibition of drug transporters is one form of modulation bacterial resistance to antimicrobial drugs [21]. Effective bacterial EPIs should reduce the intrinsic resistance of bacteria to antibiotics, reverse any acquired resistance and decrease the frequency of emergence of resistant mutant strains [17]. Effective EPIs could significantly improve antibiotic efficacy by raising physiological levels of an antibiotic and act synergistically by reducing bacterial efflux [22].

Generally, the IC_50_ values of both leaf extracts were lower against *S. aureus* (Figure 2) as compared for EPI activities against *P. aeruginosa* (Figure 1). The difference in susceptibility would most likely be due to the difference in architecture of the cell wall for Gram-positive and Gram-negative bacterial species. Gram-negative bacteria are generally known to be more resistant to antibacterials due to the double membrane while Gram positive bacteria are more sensitive to antibacterial agents because of the single layer which is not too difficult to penetrate [23–25]. However, the findings in this study showed both *P. aeruginosa* and *S. aureus* were sensitive against *V. adoensis* extract with comparable IC_50_ values of 1.64 mg/ml and 1.61 mg/ml, respectively. On the other hand, *S. aureus* was even more sensitive against *C. citrinus* with an IC_50_ value of 1.44 mg/ml, than *P. aeruginosa* which had an IC_50_ value of 1.64 mg/ml against *C. citrinus*. This could be attributed to the fact that *S. aureus* has a single membrane providing easy passage of the EPI while *P. aeruginosa* has a double membrane. These results concur with an earlier study that displayed comparable MIC values of both plant extracts against both bacteria [11].

Cytoplasmic membrane permeability was determined using the membrane-potential-sensitive cyanine dye diSC3-5. This dye is known to distribute between bacterial cells and the surrounding medium, depending on the membrane potential gradient [4]. Once inside the membrane, the dye aggregates and self-quenches. With the addition of a membrane-permeabilizing agent, dye is released and the increase of fluorescence monitored over time. In this experiment, the cell membranes of *S. aureus* and *P. aeruginosa* were loaded with the diSC3-5 dye and exposed to the plant extracts over a period of time. An increase in fluorescence was observed for both plants extracts suggesting the presence of permeabilising properties in the plant extracts. Some plant extracts have been shown in other studies to cause membrane permeability [10].

Most active plants are toxic at high doses and it is therefore important to investigate the preliminary toxicity of plant extracts [26]. This study is essential in order to determine dosage of the plant extracts which will not be lethal to the body when administered. The findings showed that haemolytic activity of extracts of the tested plants increased as concentration of the extracts arose. The increase observed was in a dose-dependent manner. According to Fick’s law, diffusion flux from a membrane is proportional to concentration difference of both sides [10]. Hence, by increasing the concentration of the extracts in extra membrane, it diffuses to intra membrane until it gets to a specific concentration, which leads to membrane destruction and haemolytic effects [10]. The haemolytic activity of the plant extracts were also shown to increase as temperature arose from 25°C to 37°C. This can be attributed to the fluidity of the bi-lipid layer of the cell membrane in which some parts of the membrane can easily move throughout the surface and this characteristic is due to membrane phospholipids which convert to jelly in temperatures lower than physiologic temperature [10]. From this study it is important to determine the levels of the leaf extracts that may be toxic to the body.

## Conclusion

Based on this study, we concluded that both *C. citrinus* and *V. adoensis* extracts have potential antibacterial activities in the form of EPI as well as cytoplasmic membrane permeabilising phytochemicals. On the other hand effective permeabilizing agents could be used to speed up the accessibility of antibiotics to targets within the bacterial cells. *C. citrinus* and *V. adoensis* leaf extracts thus have the potential to provide new lead compounds for the development of new antibiotics, efflux pump inhibitors and bacterial membrane permeabilizing agents for the treatment of *S. aureus* and *P. aeruginosa* infections. Current work is being carried out to further isolate and purify phytochemicals with antibacterial activity from both plant extracts.

### Additional information

Buffer and reagents preparation for the haemolysis assay. McIlvaine’s buffer was prepared as follows: solution 1, containing 21 g of citric acid (100 mM) and 8.775 g of sodium chloride (150 mM) made up to 1000 ml with deionized water, was mixed with solution 2, containing 28.4 g of di-sodium hydrogen phosphate (200 mM) and 8.775 g of sodium chloride (150 mM) made up to 1000 ml with deionized water, to produce the required pH of 7.0. Drabkin’s reagent was prepared by adding 1 g of sodium hydrogen carbonate, 0.2 g of potassium ferricyanide and 0.05 g of potassium cyanide in 1 litre of distilled water. Alsever solution was prepared by adding 0.05 g of glucose, 0.80 g Na-citrate and 0.42 g sodium chloride in 100 ml of bi-distilled water.

## Abbreviations

R6G: Rhodamine 6 G
diSC3-5: 3’3 dipropylthiadicarbocyanine
DMSO: Dimethylsufoxide
ATCC: American Type Culture Cell
ABC: ATP-Binding Cassette
RND: Resistance-nodulation-division
EPIs: Efflux Pump inhibitors.
